# Evaluation of the physical health of adolescent in-patients in generic and secure services: retrospective case-note review

**DOI:** 10.1192/bjb.2019.68

**Published:** 2020-06

**Authors:** Rebekah Carney, Shermin Imran, Heather Law, Siri Folstad, Sophie Parker

**Affiliations:** 1 Youth Mental Health Research Unit, Greater Manchester Mental Health NHS Foundation Trust, UK; 2 Faculty of Biology, Medicine & Health, University of Manchester, UK; 3 Child and Adolescent Mental Health Services, Greater Manchester Mental Health NHS Foundation Trust, UK

**Keywords:** Adolescent, in-patient, secure services, mental health, physical health

## Abstract

**Aims and method:**

To assess physical health needs of adolescent in-patients by routine monitoring. A retrospective analysis of case notes was conducted on a 6-month intake to generic and secure adolescent mental health units in Greater Manchester, UK.

**Results:**

Fifty individuals were admitted (52% female, average age 15.84 years). Diagnoses varied and 66% were prescribed medications before admission. All had a physical health assessment, which identified various physical health risk factors. Average body mass index was 25.99 (range 15.8–44), and increased during in-patient treatment for 84% of individuals who had their body mass recorded more than once. A total of 28% of individuals smoked. Lipids and prolactin levels were elevated across the sample.

**Clinical implications:**

This evaluation strengthens the argument to optimise physical healthcare for adolescent in-patients and develop physical health interventions, particularly given that we observed elevated lipids and prolactin. Physical health and well-being may not be prioritised when assessing and managing young peoples' mental health, despite their increased vulnerability for comorbid conditions.

People with serious mental illness (SMI) experience poor physical health, resulting in an estimated loss of life of 30 years.^1–3^ This is because of unhealthy lifestyles, medication side-effects and inadequate physical healthcare.^1,2,4^ It occurs early, often preceding the onset of illness, thus offering the opportunity to intervene to prevent comorbid conditions.^5,6^ Young people with mental health disorders are a vulnerable group requiring additional physical healthcare.^1,7^ Adolescent in-patients are particularly at risk, given the ‘obesogenic’ environment.^8,9^ This refers to additional restrictions and barriers to living a healthy lifestyle, such as reduced opportunities to exercise, lack of control over dietary intake and increased access to highly calorific snack foods. It may also be their first contact with mental health services, and affords the opportunity to intervene on an individual level through treatment access, and on a wider scale. For example, ensuring the environment encourages a healthy lifestyle and reduces the chance of iatrogenic harm. However, the precise physical health needs of adolescent in-patients is still unclear. A previous Australian study found that physical health problems are common in adolescent mental health settings, yet often go undetected and untreated.^10^ Previous evaluations suggest physical healthcare is inconsistent across services, including in-patient units, neurodevelopmental clinics and community services.^11–13^ Therefore, indicators of poor physical health are missed. However, this issue has not been evaluated in UK generic/secure adolescent in-patient services, where individuals present with a range of disorders on a variety of medications. Therefore, we aimed to evaluate the physical health needs of adolescent in-patients by routine monitoring in services in Greater Manchester, UK.

## Method

A retrospective analysis was conducted on electronic files of individuals admitted to generic and secure adolescent in-patient units over 6 months in Greater Manchester, UK (August 2018 to February 2019).

### Setting

The service is made up of units Junction 17 (generic) and Gardener (male secure). Junction 17 is a 20-bed, mixed-gender, generic adolescent in-patient service that provides various evidence-based treatments for young people with complex mental health needs. The young people admitted to the unit present with mental health symptoms that are of a severity such that they are unable to keep themselves safe, or require intensive treatment above and beyond the scope of community mental health teams. Individuals have a range of diagnoses and mental health needs, yet all are experiencing a high level of psychological distress. It also has a day service to provide alternatives to in-patient care (patients attending this were excluded from this evaluation). Referrals are received via regional child and adolescent mental health services and adult mental health services for adolescents aged 13–18 years with needs that cannot be met safely within the community. They receive intensive therapies and treatment in line with National Institute for Health and Care Excellence (NICE) guidelines and have access to a range of psychological therapies (individual and group), occupational therapy based activities and family interventions. The Gardener unit is a specialized, ten-bed, national medium secure in-patient service for adolescent males with complex health needs that cannot be met safely within the community or by standard mental health services. It is one of seven nationally commissioned services by National Health Service (NHS) England for adolescents. Referrals are received from mental health teams via a national referral process. There is also an onsite educational facility for young people provided by the Bury Educational authority. Multidisciplinary teams consist of consultant psychiatrists, psychiatric trainees and colleagues from nursing, psychology, occupational therapy, family therapy, dietetics, social work and advanced practitioners with non-medical prescribing and physical health monitoring skills.

Although ethical approval and informed consent were not sought, the evaluation was considered necessary by the clinical leads within the trust who are responsible for approving audit and service evaluations. The audit and evaluation team at Greater Manchester Mental Health NHS Foundation Trust was informed of the rationale for this evaluation and reviewed the data that was to be extracted, along with the clinicians' approval. They approved this process and provided the research team with a list of patient identification numbers to be used to extract the data. No identifiable information was extracted from the patient files and the process was conducted in line with the local guidance for service evaluations.

### Outcome measures

A structured audit tool was developed with Microsoft Excel. The following demographics were collected: gender, ethnicity, employment and living status, legal status and age on admission. Clinical variables included primary diagnoses, length of stay, discharge status and medication. Medication was recorded at two time points; the first was upon admission (this included any pre-existing prescriptions before their admission) and the second included medications prescribed at the time of discharge, or any current medications if the individual remained an in-patient at the time of the search. To maintain patient confidentiality no identifiable information was recorded.

The Physical Health Improvement Tool (PHIT) is a standardised measure developed within the service to collect physical health data for individuals upon admission. It is an electronic document that enables clinicians to record physical health assessments (blood tests, electrocardiogram (ECG), body mass index (BMI)), body composition, physical health observations, and lifestyle factors such as smoking habits, alcohol use (Alcohol Use Disorders Identification Test; AUDIT^14^), substance use, physical activity and diet, sexual health and referral to other services, e.g. sexual health services. BMI was recorded on the PHIT tool shortly after admission; some individuals also had their BMI recorded at subsequent time points, which enabled change in BMIs to be calculated.

The standard procedure for physical health monitoring is set according to the local physical health and well-being policy (Greater Manchester West Mental Health NHS Foundation Trust Physical Health and Wellbeing Policy, 2011).^15^ The policy recommends a PHIT^16^ assessment within 24 h of admission, and all individuals are to be offered appropriate lifestyle interventions in line with NICE guidelines.^7^ The PHIT tool enables clinicians to select whether individuals were offered lifestyle interventions such as smoking cessation, diet and exercise advice, and referral to drug, alcohol and sexual health services.

### Data extraction and analysis

A manual search of patient files was conducted from 12 to 19 February 2019 for the previous 6-month intake across generic and secure services (August 2018 to January 2019). Descriptive analysis was conducted with SPSS version 22 for Windows (IBM Corp., 2013) and Microsoft Excel 2016 for Windows.^17^

## Results

### Sample

Fifty individuals were admitted between August 2018 and January 2019 to the generic and secure services (52%, *n* = 26 females, 76% White British; Table 1). The average age at admission was 15.84 (range 13–21, s.d. 1.46) years. Average length of stay was 49 (range 2–169, s.d. 44.1) days. Nine (18%) were detained under the Mental Health Act 2007.^17^

**Table 1:** Demographic information

In-patient admissions	*n*	%
Junction 17	45	90%
Gardener	5	10%
Gender		
Male	24	48%
Female	26	52%
Ethnicity		
White British	38	76%
Any other White background	3	6%
Other ethnic group, Chinese	1	2%
Mixed, any other mixed background	1	2%
Other ethnic group, any other	1	2%
Black/Black British, any other	1	2%
Black/black British, African	2	4%
Employment status		
Education/training	35	70%
Unemployed	4	8%
Not recorded/stated/unknown	11	22%
Living status		
Lives with parents	5	10%
Not recorded	45	90%
Legal status		
1983 MHA section 47/49	1	2%
1983 MHA section 3	6	12%
1983 MHA section 2	2	4%
Informal	7	14%
None	34	68%
Discharge outcome		
Remains current in-patient	17	34%
Discharged	33	66%
Diagnoses		
Depressive/mood disorders	9	18%
Adjustment disorders	8	16%
Mixed anxiety and depressive disorders	7	14%
Null/not recorded	6	12%
Autism/Asperger syndrome	4	8%
Attention disturbances	3	6%
Psychotic disorders	3	6%
Conduct disorders	3	6%
Eating disorders	2	4%
Anxiety disorders	2	4%
Obsessive–compulsive disorder	1	2%
Learning difficulties	1	2%
Intentional feigning of symptoms	1	2%

MHA, Mental Health Act 2007.

### Diagnoses and medication

Individuals had a range of diagnoses, primarily mood disorders (e.g. moderate depressive episode) (*n* = 9, 18%), adjustment disorders (*n* = 8, 16%) and mixed anxiety/depressive disorders (*n* = 7, 14%) (see Table 1 for full list of diagnoses). Thirty-three (66%) were prescribed medication on admission and 32 (64%) received medication upon discharge or at the time of the search. A total of 38% (*n* = 19) were prescribed antidepressants, 18% (*n* = 9) were prescribed antipsychotics and 2% (*n* = 1) were prescribed anxiolytics at discharge or time of the search. A range of non-psychotropic medications were also prescribed to individuals for physical comorbidities such as digestive complaints and dermatological issues (see Supplementary Tables 1 and 2 available at https://doi.org/10.1192/bjb.2019.68).

### Physical health assessments

All new admissions received a physical health assessment, recorded on the electronic records with the PHIT tool. However, the extent to which the assessments were completed in full varied for each variable, and some information was missing at the time of the search either because of a delay in paperwork processing or patient or staff non-adherence.

#### Body composition

A large variation in BMI was observed at the initial physical health assessment (range 15.8–44, *n* = 44, 88%). The average BMI fell in the overweight category (mean = 25.99), and some individuals were morbidly obese (for example, two individuals had BMI values as high as BMI = 44, BMI = 35). Twenty-five (57%) individuals were within the healthy weight range, whereas seven (16%) were overweight and eight (18%) were obese or extremely obese. Twenty-six individuals had BMI recorded more than once; 84% of these individuals gained weight during this time (*n* = 21), with an average increase in BMI of 1.33 (range 0.07–5.48).

Blood pressure ratings were recorded as an average of 126.9 mm Hg (range 92–159 mm Hg, *n* = 50) systolic and 74 mm Hg (range 46–100 mm Hg, *n* = 50) diastolic.

#### Blood results and ECG recordings

A total of 70% (*n* = 35) of individuals had blood test results available at the time of data collection (Table 2). Average levels of haemoglobin a1c, random plasma glucose and cholesterol were within the healthy range for the general population; however, all individuals had elevated levels of prolactin. During times of stress, prolactin levels can reach 200 mmol/L in the general population.^18^ In this sample, 50% had prolactin levels even higher than this, displaying evidence of hyperprolactinaemia (*m* = 253.1 mmol/L, *n* = 32). A total of 87% had elevated lipid levels above the healthy average of 1 mmol/L (*m* = 1.45, range 0.8–3.5 mmol/L) and 16% had elevated triglycerides (>1.7 mmol/L), ranging up to a maximum value of 3.9 mmol/L and an average value of 1.13 mmol/L. This can be a common side effect in relation to psychotropic medication, as well as unhealthy diet.^19^ Additionally, none of the individuals who had undergone an ECG required further intervention (*n* = 30, 66%).

**Table 2:** Physical health assessments

	Average, mean (s.d.)	Range	Completed *n* (%)	Not reported, *n* (%)
PHIT				
Time between admission and PHIT assessment (days)^a^	0.35 (0.6)	0–2	50 (100%)	–
Time between admission and physical exam (days)	1 (1.74)	0–7	23 (66%)	17 (34%)
Physical health assessments				
Cardiovascular exam	–	–	30 (60%)	15 (30%)
Respiratory exam	–	–	31 (62%)	14 (28%)
Abdominal exam	–	–	30 (60%)	14 (28%)
Nervous system exam	–	–	22 (44%)	14 (28%)
ECG	–	–	30 (60%)	3 (6%)
Blood results				
Blood test results	–	–	35 (70%)	–
Haemoglobin a1c (mmol/L)	34.19 (3.80)	24–42	32 (64%)	–
Random plasma (mmol/L)	4.65 (0.75)	3–6.5	32 (64%)	–
Fasting plasma (mmol/L)	–	–	0 (0%)	–
Total random lipids (mmol/L)	3.8 (0.76)	2.3–5.6	33 (66%)	–
Random triglycerides (mmol/L)	1.13 (0.66)	0.4–3.9	32 (64%)	–
Random HDL lipids (mmol/L)	1.45 (0.54)	0.8–3.2	32 (64%)	–
Prolactin (mu/L)	253.1 (171.27)	80–955	32 (64%)	–
Body composition				
BMI	24.74 (6.69)	15.8–44.01	44 (88%)	–
Height (m)	1.67 (0.09)	1.43–1.81	45 (90%)	–
Weight (kg)	68.58 (17.36)	42.6–135	46 (92%)	–
Blood pressure, systolic	126.9 (13.2)	92–159	50 (100%)	–
Blood pressure, diastolic	74 (9.3)	46–100	50 (100%)	–

PHIT, Physical Health Improvement Tool; ECG, electrocardiogram; HDL, high-density lipoprotein; BMI, body mass index.

a. Excluding two extreme values of 7 and 20 days.

### Lifestyle

#### Physical activity and diet

As part of the PHIT assessment individuals were asked about physical activity and diet (*n* = 49, 98%). Most individuals responded to questions about consuming a diet high in fat and salt and whether they ate a balanced diet by reporting that they practiced a ‘healthy balanced diet, with no restrictions’. However, this assessment may have contradicted other available information from some healthcare professionals. For example, individuals were frequently described as being overweight, consuming a poor diet and being inactive. Precise physical activity measurements could not be obtained as individuals were asked to self-report whether they lived a sedentary lifestyle, and to describe their levels of activity. A total of 68% of individuals were offered lifestyle interventions, including weight management, advice on physical activity and diet.

#### Smoking and alcohol use

Smoking rates were higher than the general population as 28% currently smoked, compared with the average of 12% for young people in the UK.^20^ The amount of cigarettes smoked daily varied (*m* = 11, range 2–40, *n* = 7) and two individuals reported the age they started smoking (9 and 11 years). Nine smokers used cigarettes (64%) and three used roll-ups (21%). Six (42% smokers) individuals received nicotine replacement therapy as part of their routine care (Table 3).

**Table 3:** Lifestyle assessments

Physical lifestyle assessment	Yes, *n* (%)	No, *n* (%)	Not reported, *n* (%)
Reports a diet high in fat and salt	–	30 (60%)	20 (40%)
Lives a sedentary lifestyle	8 (16%)	42 (84%)	–
Aware of the risks of a sedentary lifestyle	44 (88%)	6 (12%)	–
Referral made to physical activity advice	1 (2%)	18 (36%)	31 (62%)
Smoking status			
Non-smoker (history unknown)	23 (46%)		
Current smoker	14 (28%)		
Never smoked	10 (20%)		
Ex-smoker	3 (6%)		
Alcohol use	Completed, *n* (%)	Mean (s.d.)	Range
Units of alcohol consumed per week	49 (98%)	1.02 (4.44)	0–30
AUDIT total	49 (98%)	0.81 (2.40)	0–14
Frequency of alcohol consumption	Yes, *n* (%)		
Weekly (4 or more)	1 (2%)		
Weekly (2–3 times)	1 (2%)		
Monthly (2–4 times)	4 (8%)		
Monthly or less	3 (6%)		
Never	40 (80%)		
Not recorded	1 (2%)		
Quantity of drinks on typical day			
None	40 (80%)		
1 or 2	4 (8%)		
3 or 4	3 (6%)		
10 or more	1 (2%)		
Not recorded	2 (4%)		
Times consumed ≥6 (female) or ≥8 (male) drinks on a single occasion			
Daily or almost daily	1 (2%)		
Weekly	1 (2%)		
Monthly	0		
Less than monthly	4 (8%)		
Never	42 (84%)		
Not recorded	2 (4%)		
Substance use			
Yes	6 (12%)		
Yes previous	3 (6%)		
No	39 (78%)		
Not recorded	2 (4%)		

AUDIT, Alcohol Use Disorders Identification Test.

As part of the initial PHIT assessment individuals were screened for alcohol use with the AUDIT tool.^14^ Alcohol consumption was low, and most individuals abstained (*n* = 40, 80%). Individuals were also screened for substance use (*n* = 48, 96%). Six (12%) used substances on admission and three (6%) used substances previously, including cannabis, cocaine, ketamine, LSD and aerosols.

#### Sexual health

Sexual health was discussed with ten individuals. This included whether they practiced safe sex (*n* = 10, 20%) or used contraception (*n* = 9, 18%). For females, relatively few files contained information on human papillomavirus vaccination status (*n* = 9, 35%), whether they experienced amenorrhoea (*n* = 6, 23%) or if they were pregnant (*n* = 5, 19%; no pregnancies). For males, the presence of symptoms such as erectile dysfunction were discussed with some individuals (*n* = 5, 21%). One referral was made to sexual health services.

## Discussion

The entire sample received routine physical health monitoring, and multiple various health recordings were conducted as part of these assessments. Individuals had a range of diagnoses, and displayed evidence of physical health issues requiring some form of assessment, monitoring and intervention. This is consistent with adult in-patient populations. Individual risk factors for poor physical health included high levels of obesity upon admission, subsequent weight gain, high levels of self-reported sedentary behaviour, increased smoking rates and some evidence of increased levels of lipids and prolactin. For some young people prescription of medication with metabolic side-effects included increased sense of hunger. Therefore, some of the antipsychotic medications may also be a risk factor, although only 18% of young people were prescribed antipsychotic medications in this cohort. Further information is needed to establish the dietary intake of young people and there is a need to introduce standardised measures for physical activity and diet. This evaluation highlights the vulnerability of young people admitted to in-patient wards and emphasises the opportunity this presents for physical health to be monitored, assessed and treated routinely. Although many risk factors for physical health may predate the admission, contact with health professionals during an in-patient stay affords the opportunity for healthcare provision.

### Clinical implications of findings

Our findings have important clinical implications for adolescent in-patient settings.

#### The ‘obesogenic’ environment

The ‘obesogenic’ environment of in-patient wards has frequently been discussed in the literature.^8,9^ This has been attributed to higher energy intake through increased access to high-calorie foods, reduced energy expenditure through inactivity and fewer opportunities to engage in exercise.^8,9^ Our evaluation adds further evidence to this as individuals had high BMI values, which rapidly increased with duration of stay. Weight gain in mental health services is often attributed to side-effects of antipsychotic medication; however, only a small proportion of young people were prescribed antipsychotics and those who were not also gained weight.

Unhealthy lifestyles were often reported by the clinicians. Although many received advice on living a healthy lifestyle, research has consistently shown that advice alone is insufficient to result in meaningful behaviour change.^21–23^ Clinicians should be aware of using proactive approaches to implementing lifestyle interventions and encouraging uptake of routinely offered physical health activities, such as occupational therapy groups (e.g. walking). People with SMI experience significant barriers to living healthily, such as low mood and anxiety, poor motivation, lack of social support, reduced opportunity, lack of knowledge and skills, financial barriers and employment difficulties.^24,25^ This group also has additional restrictions of being on secure and adolescent in-patient wards, living in a contained environment with relatively reduced access to facilities and outdoor opportunities. Therefore, interventions taking these additional barriers into account need to be explored, and these difficulties should be considered when attempting to promote health and well-being in this setting.

Because of the limited data available on food intake on the in-patient wards, we were unable to assess the adolescents' diet. At the time of this evaluation, routine dietary assessments were not yet conducted upon intake. This is an important and valuable opportunity to collect information on young people's eating habits and identify appropriate interventions to promote healthier diets. Systematic ways of recording food choices and dietary intake will need to be developed to facilitate this process. This could include simple charting of meal options on patient files, and conducting routine diet assessments with individuals, such as 24-hour recall to include any other foods consumed outside of regular mealtimes. Monitoring of diet will allow appropriate interventions to be targeted to those who are most in need to prevent the likelihood of weight gain.

#### Other physical health issues

Many patients were prescribed medications to alleviate physical health problems upon admission. There was also evidence of dysregulated blood metabolites and elevated levels of prolactin in over half of this sample, which is common in people with SMI.^26^ Hyperprolactinaemia can have serious consequences, such as hormonal disturbances causing sexual dysfunction, facial hair and acne, disruption to usual pubertal development in young people and increased risk of developing cancers such as breast cancer.^26–28^ This is addressed appropriately within the service and monitoring of bloods is conducted routinely. It is important for clinicians and healthcare teams to maintain routine monitoring of blood metabolites and endocrine markers as there are often no obvious symptoms to indicate individuals are at risk. Ensuring blood tests are conducted routinely, regardless of medication or diagnosis, is important. Additionally, information about sexual health screening was variable, and at the time of the search only 10% had discussions about their sexual health. This is a common issue across adolescent services. For example, a previous review found that only 37% of young people had sexual health screening upon admission to an in-patient unit.^29^ This represents a missed opportunity for management of sexual health in a high-risk group.

#### Importance of monitoring

We add to the growing evidence that physical health monitoring in mental healthcare is necessary, particularly for adolescents.^11–13,30–32^ Previous research also shows that metabolic abnormalities are common in adolescents receiving mental healthcare, but often go unnoticed and untreated.^6,10,13,21^ Individuals admitted to generic and secure mental health wards have a wide range of difficulties and non-specific mental health needs, and may or may not be prescribed psychotropic medication. It is therefore important to develop clear guidelines and policies that focus on adolescents in mental healthcare, regardless of their diagnosis or physical health status. Senior clinicians should acknowledge this when developing the standard operating procedures for their units and ensure that physical health is a fundamental part of individuals care when staying on adolescent in-patient units.

### Recommendations and future work

There is a pervasive need to explore health interventions for this group and identify the best way to deliver these within in-patient settings. Future work should focus on developing physical interventions to reduce the cardiometabolic risk associated with the in-patient environment. Hayes *et al* reviewed non-pharmacological interventions delivered on in-patient wards and found psychosocial programmes, such as therapy-based activities, family interventions and mindfulness-based activities, were common.^32^ Yet, few studies have been conducted offering physical health interventions within this setting, or even those across the general population, despite the benefits of exercise for adolescents.^33^ Further, a recent review showed that despite being recommended by NICE, lifestyle interventions are not consistently offered across mental health trusts in the UK.^34^ Standardised guidance also needs to be developed to guide clinicians and ensure adolescents are receiving high-quality physical healthcare regardless of diagnosis and in-patient status. This includes introducing formal assessments of diet and physical activity to better quantify adolescents' needs. Ensuring access to interventions alongside continued monitoring of physical heath is imperative to improving outcomes for adolescents.

### Strengths and limitations

To date, this is the first evaluation of adolescent in-patient generic and secure services that assesses routine monitoring of physical health. The findings carry significant implications for service development. This work has only been made possible because of the high levels of work happening within the unit to record all of this data, and the importance placed on ensuring the physical health assessments are conducted for all young people on admission to the units. However, this clinical audit taken from a cross-section of this population is only representative of one specific area, and the trans-diagnostic nature of the service means that physical health issues identified may change over time given the rapid turnover of young people, particularly within generic in-patient services. However, it is likely that the issues and difficulties identified here will be prevalent across mental health trusts. There is potential that some assessments identified as missing had indeed been conducted, and the data may have been uploaded after the files were searched. There is also potential for the data to exist in paper format within the service, or exist elsewhere in the electronic files rather than the physical health tool, thus resulting in some missing data. As with all routinely collected measures, they are subject to human error and reliant on accuracy of the clinicians completing the forms.

In conclusion, this evaluation strengthens the argument for optimising physical healthcare for adolescent in-patients. Adolescents admitted to generic and secure in-patient services show increased cardio-metabolic risk in the form of weight gain, obesity and dysregulated blood metabolites. We suspect that our findings are not unique to this unit and there is a need to consider physical health in adolescent in-patient services across the UK. There is a need to implement standardized routine monitoring guidelines for physical healthcare for adolescent in-patients, given their increased vulnerability, and also develop appropriate interventions in collaboration with young people to tackle the physical health disparities experienced by this group.

## Data Availability

The data included in this manuscript was extracted from routinely collected data from within the service. The data is available on secure NHS servers and, for the purpose of this study, no identifiable information was collected.

## Supplementary material

For supplementary material accompanying this paper visit https://doi.org/10.1192/bjb.2019.68. click here to view supplementary material

## About the authors

**Rebekah Carney** is a Research Associate at the Youth Mental Health Research Unit Greater Manchester Mental Health NHS Foundation Trust; and Research Associate with the Child and Adolescent Mental Health Services, Greater Manchester Mental Health NHS Foundation Trust, UK. **Shermin Imran** is a Lead Consultant Psychiatrist with the Child and Adolescent Mental Health Services, Greater Manchester Mental Health NHS Foundation Trust, UK. **Heather Law** is a Trial Manager at the Youth Mental Health Research Unit, Greater Manchester Mental Health NHS Foundation Trust, UK. **Siri Folstad** is a Research Assistant at the Faculty of Biology, Medicine & Health, University of Manchester, UK. **Sophie Parker** is a Clinical Psychologist and Director at the Youth Mental Health Research Unit Greater Manchester Mental Health NHS Foundation Trust, UK.
