# Supplementary material for: Evaluation of the physical health of adolescent in-patients in generic and secure services: retrospective case-note review
Source: BJPsych Bull. 2020 Jun;44(3):95–102. doi: 10.1192/bjb.2019.68 (PMC8058882; doi:10.1192/bjb.2019.68)
Supplement: Supplementary file 1 [file S2056469419000688sup001.docx]

**Supplementary Data**

| **Supplementary Table 1: Additional Demographics** | | | |
| --- | --- | --- | --- |
| **DISABILITY** | **Yes (*n*, %)** | **No (*n*, %)** | **Not Reported (*n*, %)** |
| Multiple Disabilities | 2 (4%) | 14 (28%) | 34 (68%) |
| Behavioural or Emotional Disability | 4 (8%) | 12 (24%) | 34 (68%) |
|  |  |  |  |
| **PHYSICAL HEALTH** |  |  |  |
| GP Details Provided | 48 (96%) | 2 (4%) | **-** |
|  |  |  |  |
| **DENTAL HEALTH** |  |  |  |
| Registered with a dentist | 41 (82%) | - | 9 (18%) |
| Check-up within past 12 months | 1 (2%) | 2 (4%) | 47 (94%) |
|  |  |  |  |
| **OPTICAL HEALTH** |  |  |  |
| Requires contact lenses or glasses | 3 (6%) | 32 (64%) | 15 (30%) |
| Issues with vision | 3 (6%) | 32 (64%) | 15 (30%) |
| Retinal Screening Required |  |  | 50 (100%) |
|  |  |  |  |
| **REFERRAL TO OTHER SERVICES** |  |  |  |
| VTE | 0 | 43 (86%) | 7 (14%) |
| Antenatal care | 0 | 4 (8%) | 46 (92%) |
| Smoking Cessation | 1 (2%) | 1 (2%) | 48 (96%) |
| Alcohol Misuse | 0 | 5 (10%) | 45 (90%) |
| Hepatitis C Testing | 0 | 15 (30%) | 35 (70%) |
| Substance Misuse | 0 | 17 (34%) | 33 (66%) |
| Cancer Screening | 0 | 15 (30%) | 35 (70%) |
| Sexual Health Services | 1 (2%) | 7 (14%) | 42 (84%) |
| Physical Activity/Lifestyle Support | 1 (2%) | 18 (36%) | 31 (62%) |
| Dietitian* | 13 (26%) |  |  |
|  |  |  |  |
| **JOB ROLE OF PHIT ASSESSOR** |  |  |  |
| Assistant Practitioner | 19 (38%) |  |  |
| Senior Nurse Practitioner | 10 (20%) |  |  |
| Nurse Practitioner | 9 (18%) |  |  |
| Registered Mental Nurse | 9 (18%) |  |  |
| Medical Secretary | 1 (2%) |  |  |
| Doctor | 1 (2%) |  |  |
| Ward Manager | 1 (2%) |  |  |

| **Supplementary Table 2: Medication Information** | | |
| --- | --- | --- |
| **Medication type (range)** | **Medication on Admission *n* (%)** | **Medication on discharge/time of search *n* (%)** |
|  |  |  |
| **Antipsychotics** |  |  |
| Risperidone (4 mg) | 1 | 1 |
| Ariprazole (5 mg – 25 mg) | 3 | 3 |
| Quetiapine (25 mg – 100 mg) | 4 | 5 |
| TOTAL | **8** | **9** |
|  |  |  |
| **Antidepressants** |  |  |
| Mirtazipine (15 mg – 45 mg) | 1 | 1 |
| Fluoxetine (20 mg – 40 mg) | 9 | 9 |
| Citalopram (30 mg – 30 mg) | 0 | 2 |
| Sertraline (50 mg – 200 mg) | 7 | 6 |
| Clomipramine (75 mg) | 0 | 1 |
| TOTAL | **17** | **19** |
|  |  |  |
| **Anxiolytic** |  |  |
| Diazepam (5 mg) | **1** | **1** |
|  |  |  |
| **BD** |  |  |
| Semisodium Valproate^a^ | **1** | **0** |
|  |  |  |
| **Vitamin/Mineral Supplements** |  |  |
| Ferrous Fumerate (210 mg) | 0 | 1 |
| Colecalceferol^a^ | 0 | 1 |
| TOTAL | **0** | **2** |
|  |  |  |
| **Physical co-morbidities** |  |  |
| *e.g. acid, gastro, digestive, skin* |  |  |
| Lactulose (15 ml) | 0 | 1 |
| Ranitidine (150 mg) | 0 | 1 |
| Asprin (75 mg) | 1 | 1 |
| Lisinopril (7.5 mg – 10 mg) | 1 | 1 |
| Omeprazole (20 mg) | 1 | 1 |
| Fostair^a^ | 1 | 0 |
| Acetazolamide (250 mg) | 1 | 1 |
| Diprobase^a^ | 0 | 1 |
| Promethezine (25 mg) | 1 | 0 |
| Lymecycline (408 mg) | 0 | 1 |
| TOTAL | **6** | **8** |
|  |  |  |
| **Contraceptives** |  |  |
| Microgynon^a^ | 1 | 2 |
| Desogesterel (75 mcg) | 1 | 2 |
| TOTAL | **2** | **4** |
|  |  |  |
| **Other** | 7 | 5 |
| Melatonin (2 mg – 6 mg) | 1 | 2 |
| Elvanse (40 mg) | **8** | **7** |

^a^ Value not provided
